# Association of Receipt of Paycheck Protection Program Loans With Staffing Patterns Among US Nursing Homes

**DOI:** 10.1001/jamanetworkopen.2023.26122

**Published:** 2023-07-27

**Authors:** Jasmine L. Travers, Brian E. McGarry, Steven Friedman, Louisa W. Holaday, Joseph S. Ross, Leo Lopez, Kevin Chen

**Affiliations:** 1 Rory Meyers College of Nursing, New York University Grossman School of Medicine, New York; 2 Division of Geriatrics and Aging, Department of Medicine, University of Rochester Medical Center, Rochester, New York; 3 Department of Population Health, New York University Grossman School of Medicine, New York; 4 Division of General Internal Medicine, Department of Medicine, Icahn School of Medicine at Mount Sinai, New York, New York; 5 Institute for Health Equity Research, Icahn School of Medicine at Mount Sinai, New York, New York; 6 Section of General Medicine and the National Clinician Scholars Program, Department of Internal Medicine, Yale School of Medicine, New Haven, Connecticut; 7 Department of Health Policy and Management, Yale School of Public Health, New Haven, Connecticut; 8 Center for Outcomes Research and Evaluation, Yale–New Haven Hospital, New Haven, Connecticut; 9 Institute for Public Health, University Health, University Medicine Associates, San Antonio, Texas; 10 Office of Ambulatory Care and Population Health, New York City Health + Hospitals, New York; 11 Division of General Internal Medicine and Clinical Innovation, Department of Medicine, New York University Grossman School of Medicine, New York

## Abstract

**Importance:**

Staffing shortages in nursing homes (NHs) threaten the quality of resident care, and the COVID-19 pandemic magnified critical staffing shortages within NHs. During the pandemic, the US Congress enacted the Paycheck Protection Program (PPP), a forgivable loan program that required eligible recipients to appropriate 60% to 75% of the loan toward staffing to qualify for loan forgiveness.

**Objective:**

To evaluate characteristics of PPP loan recipient NHs vs nonloan recipient NHs and whether there were changes in staffing hours at NHs that received a loan compared with those that did not.

**Design, Setting, and Participants:**

This economic evaluation used national data on US nursing homes that were aggregated from the Small Business Administration, Nursing Home Compare, LTCFocus, the Centers for Medicare & Medicaid Services Payroll Based Journal, the Minimum Data Set, the Area Deprivation Index, the Healthcare Cost Report Information System, and the US Department of Agriculture Rural-Urban Continuum Codes from January 1 to December 23, 2020.

**Exposure:**

Paycheck Protection Program loan receipt status.

**Main Outcome and Measures:**

Staffing variables included registered nurse, licensed practical nurse (LPN), and certified nursing assistant (CNA) total hours per week. Staffing hours were examined on a weekly basis before and after loan receipt during the study period. An event-study approach was used to estimate the staffing total weekly hours at NHs that received PPP loans compared with NHs that did not receive a PPP loan.

**Results:**

Among 6008 US NHs, 1807 (30.1%) received a PPP loan and 4201 (69.9%) did not. The median loan amount was $664 349 (IQR, $407 000-$1 058 300). Loan recipients were less likely to be part of a chain (733 [40.6%] vs 2592 [61.7%]) and more likely to be for profit (1342 [74.3%] vs 2877 [68.5%]), be located in nonurban settings (159 [8.8%] vs 183 [4.4%]), have a greater proportion of Medicaid-funded residents (mean [SD], 60.92% [21.58%] vs 56.78% [25.57%]), and have lower staffing quality ratings (mean [SD], 2.88 [1.20] vs 3.03 [1.22]) and overall quality star ratings (mean [SD], 3.08 [1.44] vs 3.22 [1.44]) (*P* < .001 for all). Twelve weeks after PPP loan receipt, NHs that received a PPP loan experienced a mean difference of 26.19 more CNA hours per week (95% CI, 14.50-37.87 hours per week) and a mean difference of 6.67 more LPN hours per week (95% CI, 1.21-12.12 hours per week) compared with nursing homes that did not receive a PPP loan. No associations were found between PPP loan receipt and weekly RN staffing hours (12 weeks: mean difference, 1.99 hours per week; 95% CI, −2.38 to 6.36 hours per week).

**Conclusions and Relevance:**

In this economic evaluation, a forgivable loan program that required funding to be appropriated toward staffing was associated with a significant increase in CNA and LPN staffing hours among NH PPP loan recipients. Because the PPP loans are temporary, federal and state entities may need to institute sufficient and sustainable support to mitigate NH staffing shortages.

## Key Points

**Question:**

Did staffing hours change among nursing homes that received forgivable loans through the Paycheck Protection Program (PPP), which required recipients to appropriate 60% to 75% of the loan toward staffing?

**Findings:**

In this economic analysis of 6008 US nursing homes, 12 weeks after PPP loan receipt, nursing homes that received a PPP loan experienced increases in certified nursing assistant hours per week and licensed practical nurse hours per week compared with nursing homes that did not receive a PPP loan. No association was found between PPP loan receipt and registered nurse staffing hours per week.

**Meaning:**

The findings suggest that dedicated funding programs for staffing may be associated with increased nursing home staffing hours.

## Teaser

This economic evaluation assesses the association between Paycheck Protection Program loan receipt and nursing home staffing hours.

## Introduction

The COVID-19 pandemic magnified several long-standing problems within nursing homes (NHs), including critical staffing shortages.^1^ In March 2020, at the start of the pandemic, more than 1 in 5 NHs in the US reported severe staffing shortages.^1^ The most commonly reported staff shortage was in nurse aides (18.4%), licensed nurses (15.9%), clinical staff (2.5%), and other staff (9.8%).^2^ Staffing shortages in NHs have threatened the quality of resident care significantly, especially when these shortages have occurred among frontline care staff.^1,2^

Nursing homes with a greater proportion of Medicare residents were less likely to experience staffing shortages.^2^ Compared with Medicaid, which often pays less than reported operating costs, Medicare provides higher reimbursement to NHs, allowing for more investment in staff and quality.^2,3^ This reimbursement plan suggests that the funding NHs receive is directly associated with staffing efforts.^2^ As such, stakeholders have advocated for increased reimbursement to NHs; however, NH advocacy groups, such as LeadingAge, have warned against simply raising funds for NHs without ensuring that they will go toward areas important to resident care, such as staffing.^4^

To provide a solution that ensures that staffing receives adequate investment, a number of states, federal entities, and most recently, the National Academies of Sciences, Engineering, and Medicine have called for specified proportions of NH funding (eg, Medicare and Medicaid payments) to be appropriated toward staffing.^5^ Information is still needed on what percentage of funding would be sufficient to appropriate toward staffing levels as well as whether and how appropriation is associated with NH staffing levels. In a recent study on NH spending and staffing levels, Bowblis et al^6^ identified that the median proportion of revenues spent on nursing staff in 2019 was 33.9%. When examining staffing levels across a threshold of 25% to 45% of revenues, the authors found that facilities with higher shares of Medicaid residents spent a larger share of revenues on nursing staff but had lower staffing levels. That study, however, was cross-sectional and only reported NHs as having thresholds of appropriation between 25% to 45%, whereas levels of 60% to 75% have been proposed as being ideal.

One opportunity for examining how appropriated funding at a higher level may affect staffing is through the Paycheck Protection Program (PPP). The PPP was a loan program to support small businesses, including NHs, during the COVID-19 pandemic in the form of providing a direct incentive for small businesses to keep their workers on payroll. The loan was forgivable if recipients appropriated at least 60% to 75% of the loan they received toward payroll and staffing.^7^ Assessing the PPP may inform how appropriating a specified proportion of funding toward staffing efforts might influence staffing levels.

We sought to understand the characteristics of NHs that received PPP loans and the changes in staffing levels (ie, hours) after they received the loans compared with NHs that did not receive PPP loans. These findings may help policy makers better understand whether the PPP may be a model for future programs seeking to appropriate specific funds to NH staffing.

## Methods

### Design

This economic evaluation used a difference-in-differences event-study design to estimate the association of PPP loan receipt with staffing hours at NHs that received a PPP loan compared with NHs that did not receive a PPP loan. With this approach, we were able to account for other local, state, and federal efforts that may have been occurring at the time when the PPP loans were available, providing a natural experiment. This study met predetermined criteria for institutional review board exemption according to the Common Rule due to the use of deidentified data. This study followed the Strengthening the Reporting of Observational Studies in Epidemiology (STROBE) reporting guideline and relevant parts of the Consolidated Health Economic Evaluation Reporting Standards (CHEERS) reporting guideline.^8^

### Data Sources

Eight data sources were used for this study: the Small Business Administration (SBA),^9^ Nursing Home Compare,^10^ LTCFocus,^11^ the Centers for Medicare & Medicaid Services (CMS) Payroll Based Journal,^12^ the Minimum Data Set (MDS),^13^ the Area Deprivation Index,^14^ the skilled nursing facility cost data set at the Healthcare Cost Report Information System,^15^ and US Department of Agriculture (USDA) Rural-Urban Continuum Codes.^16^ All data sources are publicly available except the MDS.

#### SBA

The SBA maintains data on all small businesses that received PPP loans, including NHs.^9^ The PPP originated under the 2020 CARES Act.^17^ Almost any small business that existed before February 15, 2020, was able to apply for a forgivable loan of up to $10 million to retain their workforce and assist with operational expenses. Nursing homes included in this data set meet the SBA definition of a small business as having fewer than 500 employees or, in the case of nursing care facilities, less than $30 000 000 in annual revenues. At least 60% to 75% of the PPP loan needed to be used for payroll costs for the loan to be forgiven. To apply for loan forgiveness, the small business had to provide documentation for all payroll periods along with nonpayroll expenses that overlapped with the covered period.^18^ Loan amounts were determined by calculating the average monthly cost of employee salaries and the loan amounts paid up to 8 weeks of payroll costs and benefits. After June 5, 2020, the default spend-down period became the shorter of 24 weeks or time remaining until December 31, 2020. Entities receiving loans before June 5 could opt to extend from 8 weeks to 24 weeks. A substantial number of skilled nursing facilities successfully applied for these funds.^19^ We focused on funds that were disbursed during the first round of the loans, which occurred from April through August 2020.

#### Nursing Home Compare

Data in Nursing Home Compare are collected quarterly and allow for identification of and comparisons between NHs that are certified by the CMS.^10^ The data set includes information about the quality of care provided by these NHs, including how they have performed on health inspections and how they are staffed, enforcement actions the government has taken against them, and how well their residents are treated in specific areas of care. Variables of interest from this data source were facility characteristics, staffing quality score, overall star rating, and ownership.

#### LTCFocus

LTCFocus is a data set organized by Brown University that provides the facility and aggregated resident characteristics of all Medicare- and Medicaid-certified NHs in the US compiled from multiple data sources.^11^ We used 2018 data, which were the most up-to-date data available at the time of our analysis.

#### CMS Payroll Based Journal

The CMS Payroll Based Journal provides reports on direct care staffing information.^12^ We used staffing hours for quarters 1 through 4 of 2020.

#### MDS

The MDS, owned by CMS, is a standardized assessment tool that measures the health status of NH residents.^13^ These assessments are performed and recorded by NH staff and include information on resident characteristics, such as race and ethnicity, functional and cognitive status, psychosocial functioning, geriatric syndromes (eg, dementia, depression), and end-of-life care wishes. Variables of interest from this data source included race and ethnicity aggregated to the facility level.

#### Area Deprivation Index

The Area Deprivation Index is a measure created as a composite index with greater validity, robustness, and explanatory power than single-area measures in documenting the extent of social disparities in health and mortality^20^ and is based on a measure created by the Health Resources & Services Administration.^21^ This index is a nationally relative measure with scores from 1 to 100 at the census-block level. Higher scores indicate greater socioeconomic disadvantage in the neighborhood. A national ranking of 85th percentile or higher was considered severely disadvantaged based on prior research. The 2018 version of the index was used in this study.^14^

#### USDA Rural-Urban Continuum Codes

USDA Rural-Urban Continuum Codes distinguish metropolitan counties by the population size of their metropolitan area and nonmetropolitan counties by degree of urbanization and adjacency to a metropolitan area.^16^ We present information on the rurality and urbanicity of the NHs.

#### Healthcare Cost Report Information System Skilled Nursing Facility Cost Data Set

Medicare-certified facilities are required to submit an annual cost report to a Medicare administrative contractor. The cost report contains institutional information, such as facility characteristics, utilization data, cost and charges by cost center (in total and for Medicare), Medicare settlement data, and financial statement data.^15^ We used these data to calculate total revenue for the NH.

### Measures

#### Demographic Variables

Demographic characteristics of the NHs that were used for analyses included the percentage of Black residents in the NH (we focused on the percentage of Black residents because other studies have found poorer staffing and resident outcomes in nursing homes with greater percentages of Black residents^22,23^), region, urbanicity (urban vs rural), Area Deprivation Index (dichotomized, ≥85th percentile [severely disadvantaged] vs <85 percentile [less disadvantaged]), staffing quality rating (range, 1 to 5; a higher rating suggests that the facility has sufficient staffing levels and a higher likelihood of providing quality care and attention to its residents), overall star rating (range, 1-5), bed size, total residents per day, percentage occupancy, ownership (for profit, nonprofit, or government), chain ownership, and percentage of Medicaid residents in the NH.

#### Outcome and Independent Variables

Our outcome variables were continuous and included staffing (registered nurse [RN], licensed practical nurse [LPN], and certified nursing assistant [CNA]) total weekly hours. Our main independent variable was the PPP loan, dichotomized as loan recipient vs nonrecipient.

### Statistical Analysis

#### Study Design

We merged the 8 data sources to conduct our analyses. We used business addresses as the linking identifier between PPP and Nursing Home Compare data. We then linked the additional data sets by the institution identification numbers, which were contained in the NH Compare data set and subsequent data sets. We first examined descriptive characteristics of the study sample using a Kruskal-Wallis test for continuous outcomes and a χ^2^ test for categorical outcomes to compare characteristics of facilities that received a PPP loan with NHs that did not. We then estimated multiple event-study models based on weekly staffing hours for the 3 staff types (RN, LPN, and CNA) using generalized linear model linear regression models that included fixed effects for each nursing home and calendar week. Using an event-study approach allowed us to estimate the association of PPP loan receipt with staffing total weekly hours compared with changes in staffing total weekly hours experienced in non–PPP recipient NHs over the same period.

#### Study Sample

Only NHs that were eligible for the PPP loan were included in our analyses (NHs with <$30 million in revenue). Additionally, staffing data were not available in the CMS Payroll Based Journal data set for the first quarter of 2020. Moreover, the median loan date was April 15, 2020. Therefore, to ensure that we were able to extend the event time to more than 2 weeks before loan receipt to assess for parallel trends, we conducted a complete case analysis for NHs that had staffing data for all quarters. For each PPP loan recipient, we identified all NHs in the same county that did not receive PPP loans during the study period. Matched neighboring facilities served as controls. We dropped any facility that was not a treatment facility or matched control. This approach allowed us to account for events that were occurring in the county that might have additionally affected staffing, such as changes in numbers of COVID-19 cases.

#### Event Time

The study period was from January 1 to December 23, 2020. Event time was defined on a weekly basis relative to the date of initial PPP loan receipt for the treatment facility. Pre-event time included all weeks that preceded the loan recipient date (week 0) during the study period. Postevent time included all weeks that followed the loan recipient date during the study period. Event time was truncated on a monthly basis between −12 weeks and 24 weeks. The reference week was week −1, immediately preceding loan receipt, to assess the difference between the control and PPP recipient group. Control facilities had the same event time as their matched treatment facility.

All analyses were conducted in SAS, version 9.4 (SAS Institute Inc). We report coefficients, 95% CIs, and *P* values. The coefficients represent the adjusted mean difference attributed to that variable. The significance level was set at 2-sided *P* < .05.

## Results

Among 6008 US NHs eligible for a PPP loan and with complete first-quarter staffing data, 1807 (30.1%) received a PPP loan and 4201 (69.9%) did not. The median loan date of PPP receipt was April 15, 2020. The median loan amount among recipients was $664 349 (IQR, $407 000-$1 058 300). Nursing homes that received a PPP loan were less likely to be part of a chain (733 [40.6%] vs 2592 [61.7%]) and more likely to be for profit (1342 [74.3%] vs 2877 [68.5%]), located in nonurban settings (159 [8.8%] vs 183 [4.4%]), have a greater proportion of Medicaid-funded residents (mean [SD], 60.92% [21.58%] vs 56.78% [25.57%]), and have lower staffing quality ratings (mean [SD], 2.88 [1.20] vs 3.03 [1.22]) and overall quality star ratings (mean [SD], 3.08 [1.44] vs 3.22 [1.44]) (*P* < .001 for all) (Table 1). At 4 weeks before the loans were issued, NHs that received a PPP loan had fewer total staffing hours (mean [SD], 2030 [1063] hours) compared with NHs that did not receive a PPP loan (mean [SD], 2176 [1236] hours). When estimating staffing hours separately at 4 weeks before the loans were issued, mean (SD) RN, LPN, and CNA hours for NHs that received the PPP loans were less (326 [235], 480 [318], and 1224 [658] hours, respectively) compared with nursing homes that did not receive the PPP loans (375 [297], 510 [330], and 1290 [775], hours respectively).

**Table 1.** Characteristics of PPP Nursing Home Loan Recipients and Nonrecipients

Characteristic	Nursing homes^a^			*P* value
	Total PPP eligible (N = 6008)	PPP loan nonrecipient (n = 4201)	PPP loan recipient (n = 1807)	
Ownership				
For profit	4219 (70.2)	2877 (68.5)	1342 (74.3)	<.001
Government	316 (5.3)	254 (6.0)	62 (3.4)	
Nonprofit	1473 (24.5)	1070 (25.5)	403 (22.3)	
Chain ownership	3325 (58.9)	2592 (61.7)	733 (40.6)	<.001
Urbanicity				
Metropolitan	4910 (81.7)	3568 (84.9)	1342 (74.3)	<.001
Nonmetropolitan				
Adjacent to a metropolitan area	756 (12.6)	450 (10.7)	306 (16.9)	
Not adjacent to a metropolitan area	342 (5.7)	183 (4.4)	159 (8.8)	
Area Deprivation Index^b^				
Less disadvantaged	5029 (85.8)	3540 (84.3)	1489 (82.4)	.20
Severely disadvantaged	831 (14.2)	560 (13.3)	271 (15.0)	
Missing	148 (2.5)	101 (2.4)	47 (2.6)	
Staffing quality rating, mean (SD)^c^	2.99 (1.22)	3.03 (1.22)	2.88 (1.20)	<.001
Overall star rating, mean (SD)	3.18 (1.44)	3.22 (1.44)	3.08 (1.44)	<.001
Black residents, %^d^				
0	1111 (18.5)	725 (21.7)	386 (26.4)	.01
<5	1488 (24.8)	1051 (31.4)	437 (29.9)	
5-19.9	1315 (21.9)	934 (27.9)	381 (26.1)	
20-49.9	643 (10.7)	457 (13.6)	186 (12.7)	
>50	252 (4.2)	181 (5.4)	71 (4.9)	
Occupancy, mean (SD), %	80.70 (14.32)	81.00 (14.39)	80.01 (14.14)	.003
Total residents per d, mean (SD), No.	82.01 (42.47)	82.35 (43.69)	81.2 (39.47)	.35
Certified beds, mean (SD), No.	101.78 (52.43)	101.47 (54.58)	102.5 (47.05)	.27
Funded by Medicaid, mean (SD), %	58.02 (24.51)	56.78 (25.57)	60.92 (21.58)	<.001

Abbreviation: PPP, Paycheck Protection Program.

^a^ Data are presented as number (percentage) of nursing homes unless otherwise indicated.

^b^ Less disadvantaged was defined as an Area Deprivation Index less than 85% and severely disadvantaged as 85% or higher.

^c^ This rating system typically assigns a numerical score ranging from 1 to 5, with 1 being the lowest and 5 being the highest staffing quality rating. A higher rating suggests that the facility has sufficient staffing levels and a higher likelihood of providing quality care and attention to its residents.

^d^ Proportions based on the 2018 Minimum Data Set.^13^

At 12 weeks after receipt of the PPP loan, recipient NHs had 34.84 more total staffing hours per week (95% CI, 17.53-52.16 hours per week) than did NHs that did not receive the PPP loans; CNA and LPN hours were 26.19 hours per week (95% CI, 14.50-37.87 hours per week) and 6.67 hours per week (95% CI, 1.21-12.12 hours per week) more, respectively, in PPP-recipient NHs than in nonrecipient NHs. While total staffing hours continued to be greater among NHs that received a PPP loan compared with NHs that did not receive a PPP loan during the post–loan receipt period, 12 weeks after receipt, the magnitude in the differences in staffing hours between the PPP recipient NHs and the nonrecipient NHs generally plateaued (Table 2 and eFigures 1-5 in Supplement 1).

**Table 2.** Event-Study Staffing Regression Estimates for Paycheck Protection Program Recipient Nursing Homes Compared With Non–Paycheck Protection Program Recipient Nursing Homes

Event time, wk	Coefficient, h (95% CI)			
	Total	RN	LPN	CNA
−12	19.95 (6.22 to 33.69)^a^	0.38 (−3.09 to 3.84)	4.85 (0.52 to 9.18)^b^	14.72 (5.45 to 24.00)^a^
−8	−13.63 (−30.80 to 3.54)	−4.99 (−9.33 to −0.66)^b^	−1.25 (−6.66 to 4.17)	−7.39 (−18.98 to 4.20)
−4	−18.99 (−35.96 to −2.02)^b^	−5.63 (−9.91 to −1.34)^b^	−0.77 (−6.12 to 4.58)	−12.60 (−24.05 to −1.14)^b^
0	3.28 (−13.69 to 20.26)	1.53 (−2.75 to 5.82)	−0.45 (−5.80 to 4.90)	2.20 (−9.26 to 13.66)
4	16.73 (−0.51 to 33.98)^c^	0.56 (−3.79 to 4.91)	2.64 (−2.79 to 8.08)	13.53 (1.89 to 25.17)^b^
8	21.72 (4.43 to 39.00)^b^	0.52 (−3.84 to 4.88)	3.67 (−1.78 to 9.12)	17.53 (5.86 to 29.19)^a^
12	34.84 (17.53 to 52.16)^d^	1.99 (−2.38 to 6.36)	6.67 (1.21 to 12.12)^b^	26.19 (14.50 to 37.87)^d^
16	38.04 (20.72 to 55.35)^d^	2.86 (−1.51 to 7.23)	6.35 (0.89 to 11.80)^b^	28.83 (17.14 to 40.52)^d^
20	40.32 (22.93 to 57.71)^d^	2.81 (−1.58 to 7.20)	11.26 (5.78 to 16.74)^d^	26.25 (14.51 to 37.99)^d^
24	43.54 (30.55 to 56.53)^d^	4.57 (1.29 to 7.85)^a^	9.88 (5.78 to 13.97)^d^	29.10 (20.33 to 37.87)^d^

Abbreviations:, CNA, certified nursing assistant; LPN, licensed practical nurse; RN, registered nurse.

^a^ *P* < .01.

^b^ *P* < .05.

^c^ *P* < .10.

^d^ *P* < .001.

## Discussion

We sought to understand whether a program that stipulated the appropriation of a specified proportion of funding toward staffing was associated with changes in staffing hours within NHs. After linking multiple data sources that include information on NH populations and quality, small businesses, and communities, we found that receipt of PPP loans was associated with increases in staffing hours for CNAs and LPNs compared with no receipt of PPP loans among PPP-eligible NHs. Nursing homes that received PPP loans had lower staffing hours before the COVID-19 pandemic and were more likely to have facility characteristics reflective of poorer quality of care compared with NHs that did not receive the loans.

It is important to put into context the median loan amount for NHs that received the PPP loan. The CARES Act stimulus package^24^ provided a baseline payment of $50 000 plus an additional $2500 per bed, which for an NH of 100 beds, would equate to $300 000. Through the PPP loan, NHs received a median loan amount of $664 349, which is about twice as much as that from the stimulus package. Regarding the cost of staffing, in 2021, the median cost of an RN was $77 600 annually and $37.31 hourly.^25^ The median cost of a CNA was $30 290 annually and $14.56 hourly.^26^ Based on these wages, the NH would have been able to pay 17 153 additional RN hours or 43 956 additional CNA hours with the loan that they received.^27^ As the NH could have paid for other payroll and nonpayroll costs, such as benefits, it is unclear how much of the PPP loan was dedicated to staffing hours.

Regarding the differences in the change in staffing hours between PPP recipients and NHs that did not receive a loan, NHs that received the PPP loan were able to provide a significantly greater number of CNA hours; these equated to 2 additional CNA shifts per week as soon as 4 weeks after the PPP loan and 4 additional CNA shifts per week at week 24 after PPP loan receipt. Regarding LPNs, NHs were able to schedule approximately 1 additional LPN shift per week at 12 weeks after PPP loan receipt.

While it was surprising that increases in RN staffing hours were not significantly different among NHs that received PPP loans for much of the period after receiving a PPP loan, there are plausible reasons for this. Among prior reports of staffing shortages, most were for nurse aides (CNAs).^1,2^ CNAs provide the most direct care to NH residents, followed by LPNs, so they are needed in greater numbers compared with other clinical staff.^2^ This highlights the importance of the CNA and LPN workforce to NH care and suggests that CNA and LPN staffing might have been where NHs receiving PPP loans prioritized their funds. Staffing of CNAs and LPNs is also less costly than staffing of RNs.^28^ Thus, when seeking to increase the hours of staff available to provide care to residents, it might have been most practical for NHs to focus on employing the greatest number of staff possible with limited funding. Lastly, it is likely that RNs were not as readily available to fill staffing shortages in NHs, as other settings, such as acute care, were experiencing shortages also and incentives to work in those settings were greater.

A similar approach to bolstering staffing and reducing vacancies and turnover through required funds toward staffing is wage pass-through laws. A wage pass-through is an additional allocation of funds provided through Medicaid reimbursement for the purpose of increasing compensation, benefits, or the number of staff for direct care workers. Twenty-one states and Washington, DC, have wage pass-through laws, in which a study found that the introduction of wage pass-throughs over the study period was associated with between 3.0% and 4.0% net increases in CNA hours per residents per day in the years following adoption.^29^ Currently, the allocation of funds for wage pass-throughs varies across states, and states can designate either a set dollar amount per staff hour (or resident day) or a certain percentage of a reimbursement increase to be used for wages and benefits. Our findings provide more information to states for understanding how allocating a certain percentage of funds may change staffing levels.

Our findings demonstrated that the NHs that were most in need of funding, as evidenced by lower quality and staffing scores, were recipients of the PPP loans. Other characteristics that have been associated with lower quality were also more prevalent among PPP loan recipients, which were more likely to be for-profit NHs. These findings reflect the importance of ensuring that NHs with facility characteristics associated with poorer quality care have access to and are aware of funds for operational support.

### Implications

Our findings have important policy and research implications, the first of which is supporting advocacy for the need to appropriate funds.^30^ While our research was unable to provide information on variation in staffing hours based on the level of appropriation, proportions that have been proposed in the past include 60% and 75%.^31^ Future research should include prediction analyses on what these changes in staffing hours might look like based on fund appropriation level. Next, while we found that receiving a PPP loan was associated with increased staffing hours, PPP loans were only temporary. There is a need for federal and state entities to institute sustainable support that incentivizes NHs to invest in their staff long term. States have incentivized NHs to do so in the form of requirements that revenues be appropriated toward staffing. New York requires that 70% of revenues go toward direct resident care, of which 40% must go toward paying staff.^32,33^ However, it is likely that NHs will need additional support to meet these requirements. Lastly, other frontline staff, such as RNs, are vital for resident care; RNs assess the residents and help decrease avoidable health service utilization, such as emergency department visits.^34^ The 2022 National Academies of Science, Engineering, and Medicine report on NHs endorses long-standing recommendations for the 24-hour presence of an RN.^35^ It is important to provide sufficient funding to fill gaps in staffing needs across positions. Looking toward future research, investigators should perform a large-scale survey or qualitative inquiry to understand how NHs used their PPP funds along with non–PPP-funded assistance and gauge their awareness of such programs.

### Limitations

There are several limitations. First, because of the absence of institution identification numbers in the PPP database and having to merge the business names and addresses of NHs therein with corresponding information in the NH Compare database, there could have been discrepancies in this approach. It is possible that we did not capture all NHs that received a loan, and/or we may have erroneously identified an NH that received a PPP loan. However, this would bias findings toward the null. Second, because NHs were not required to appropriate 60% to 75% of the PPP loan to staffing if they did not care for loan forgiveness, we do not know which NHs in our sample did so. However, nearly 100% of the loans were forgiven; therefore, it is likely that the majority of the NHs in our PPP sample appropriated requisite funds to staffing.^36^ Next, the PPP loans were intended to help with external stressors of NH staffing (ie, the pandemic), not intrinsic stressors; therefore, staffing changes potentially attributable to PPP loans may not be generalizable to expected changes for funding to support nonpandemic staffing shortages. Lastly, it would have been ideal to examine whether the receipt of PPP loans made NHs less likely to report a staffing shortage; however, these data only became available beginning May 2020, which did not allow for us to look at these data before or immediately after the PPP.

## Conclusions

This economic evaluation found that a forgivable loan program that called for 60% to 75% of funds to be appropriated toward staffing was associated with a significant increase in staffing hours for CNAs and LPNs. The increase in staffing levels may have been associated with a higher quality of care for residents, which has been supported by studies examining the association between staffing levels and resident outcomes.^37,38^ PPP loans were only temporary, however, and staffing shortages existed beyond CNAs and LPNs before and during the pandemic. Thus, there appears to be a need for federal and state entities to institute sufficient and sustainable support that incentivizes NHs to invest in all frontline staff. To our knowledge, this is the first study to assess the association of appropriated staffing funds with NH staffing hours over time.

## Notes

Supplement 1. **eFigure 1.** Change in total hours per week for all nursing staff, CNAs, RNs, and LPNs pre and post PPP loan receipt compared to non-PPP NH loan recipients **eFigure 2.** Change in total hours per week for all nursing staff pre- and post-PPP loan receipt compared to non-PPP NH loan recipients **eFigure 3.** Change in total hours per week for CNAs pre- and post-PPP loan compared to non-PPP NH loan recipients **eFigure 4.** Change in total hours per week for LPNs pre- and post-PPP loan compared to non-PPP NH loan recipients **eFigure 5.** Change in total hours per week for RNs pre- and post-PPP loan compared to non-PPP NH loan recipients

Supplement 2.
Data Sharing Statement
