# Supplementary material for: Association of Receipt of Paycheck Protection Program Loans With Staffing Patterns Among US Nursing Homes
Source: JAMA Netw Open. 2023 Jul 27;6(7):e2326122. doi: 10.1001/jamanetworkopen.2023.26122 (PMC10375300; doi:10.1001/jamanetworkopen.2023.26122)
Supplement: Supplement 1. — eFigure 1. Change in total hours per week for all nursing staff, CNAs, RNs, and LPNs pre and post PPP loan receipt compared to non-PPP NH loan recipients eFigure 2. Change in total hours per week for all nursing staff pre- and post-PPP loan receipt compared to non-PPP NH loan recipients eFigure 3. Change in total hours per week for CNAs pre- and post-PPP loan compared to non-PPP NH loan recipients eFigure 4. Change in total hours per week for LPNs pre- and post-PPP loan compared to non-PPP NH loan recipients eFigure 5. Change in total hours per week for RNs pre- and post-PPP loan compared to non-PPP NH loan recipients [file jamanetwopen-e2326122-s001.pdf]

Supplemental Online Content

Travers JL, McGarry BE, Friedman S, et al. Association of receipt of Paycheck Protection Program loans with staffing patterns among US nursing homes. *JAMA Netw Open.* 2023;6(7):e2326122. doi:10.1001/jamanetworkopen.2023.26122

eFigure 1. Change in total hours per week for all nursing staff, CNAs, RNs, and LPNs pre and post PPP loan receipt compared to non-PPP NH loan recipients

eFigure 2. Change in total hours per week for all nursing staff pre- and post-PPP loan receipt compared to non-PPP NH loan recipients

eFigure 3. Change in total hours per week for CNAs pre- and post-PPP loan compared to non-PPP NH loan recipients

eFigure 4. Change in total hours per week for LPNs pre- and post-PPP loan compared to non-PPP NH loan recipients

eFigure 5. Change in total hours per week for RNs pre- and post-PPP loan compared to non-PPP NH loan recipients

This supplemental material has been provided by the authors to give readers additional information about their work.

eFigure1. Change in total hours per week for all nursing staff, CNAs, RNs, and LPNs pre and post PPP loan receipt compared to non-PPP NH loan recipients

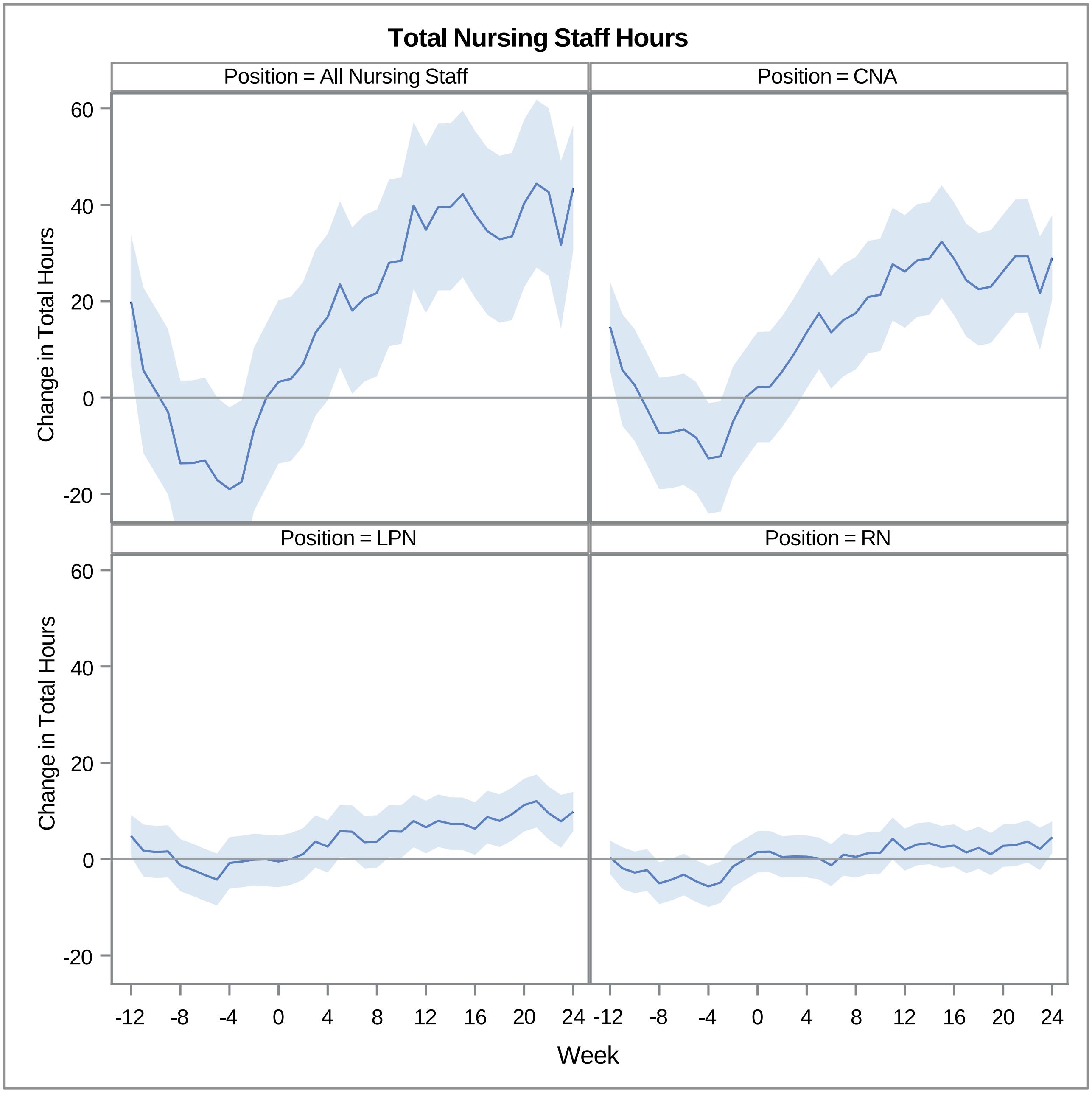

CNA=Certified Nursing Assistant, RN=Registered Nurse, LPN=Licensed Practical Nurse; PPP= Paycheck Protection Program, NH= nursing home

© 2023 Travers JL et al. *JAMA Network Open.*

eFigure2. Change in total hours per week for all nursing staff pre- and post- PPP loan receipt compared to non-PPP NH loan recipients

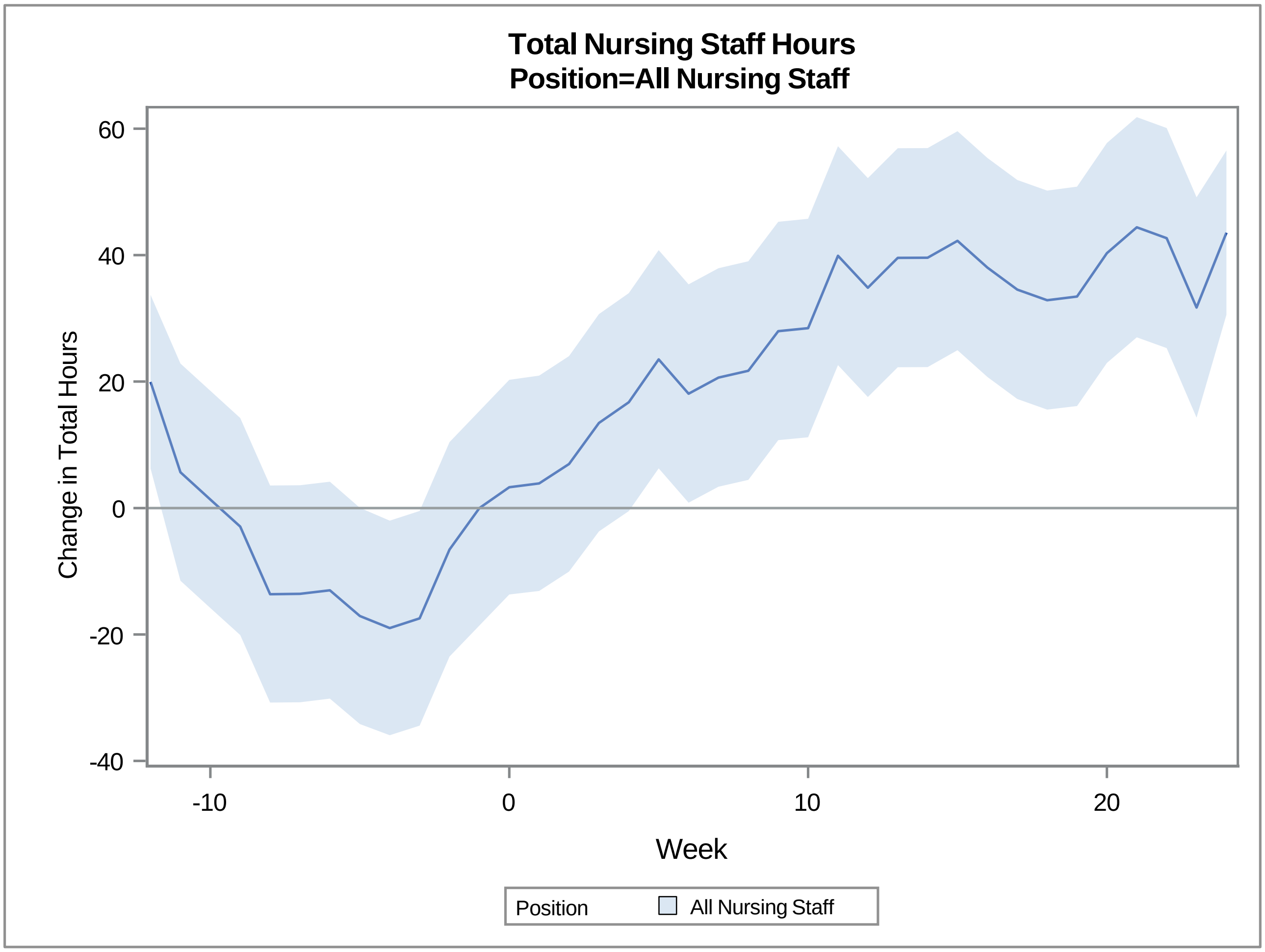

PPP= Paycheck Protection Program, NH= nursing home

© 2023 Travers JL et al. *JAMA Network Open.*

eFigure3. Change in total hours per week for CNAs pre- and post- PPP loan compared to non-PPP NH loan recipients

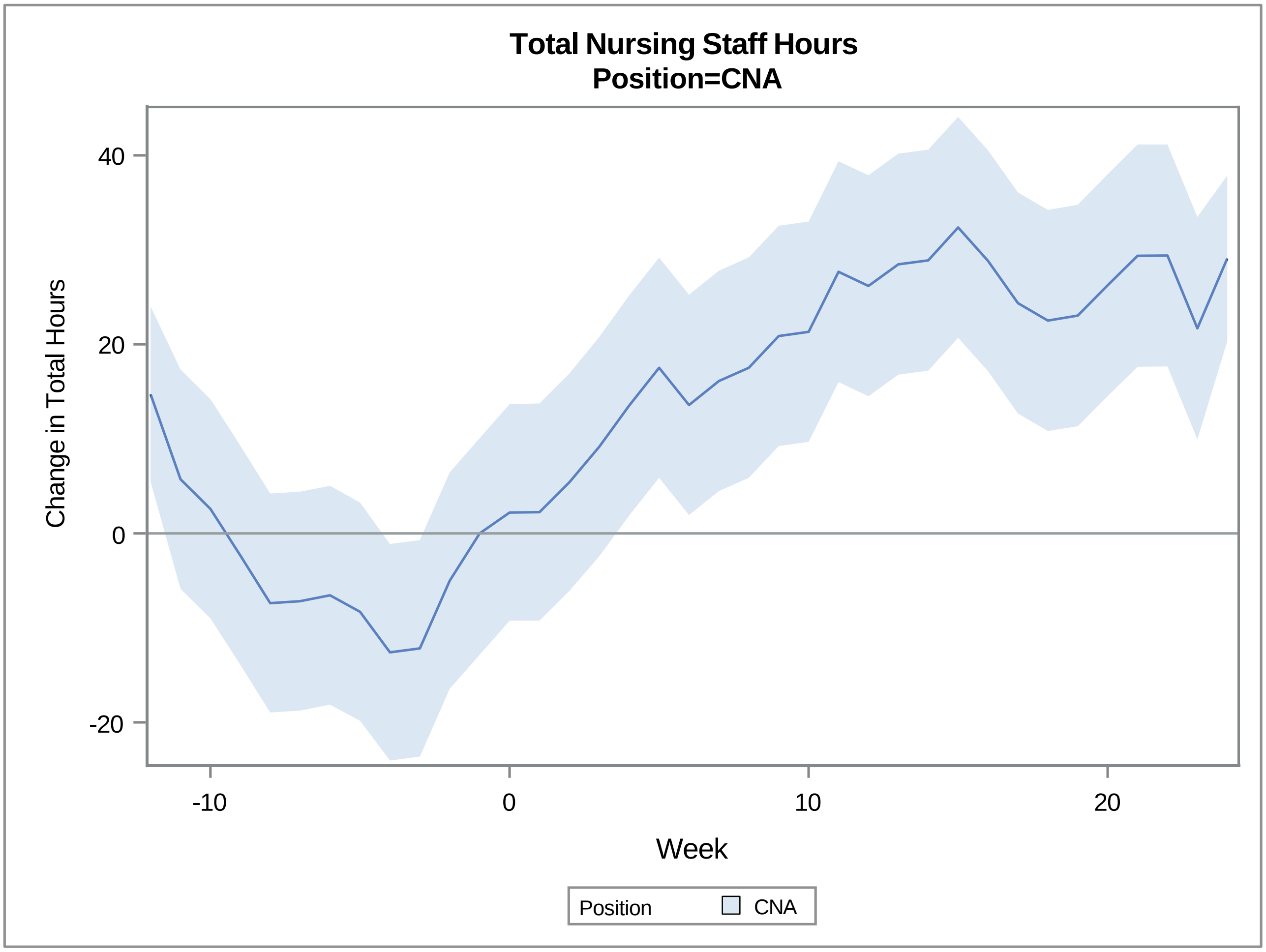

CNA= Certified Nursing Assistant; PPP= Paycheck Protection Program, NH= nursing home

eFigure4. Change in total hours per week for LPNs pre- and post- PPP loan compared to non-PPP NH loan recipients

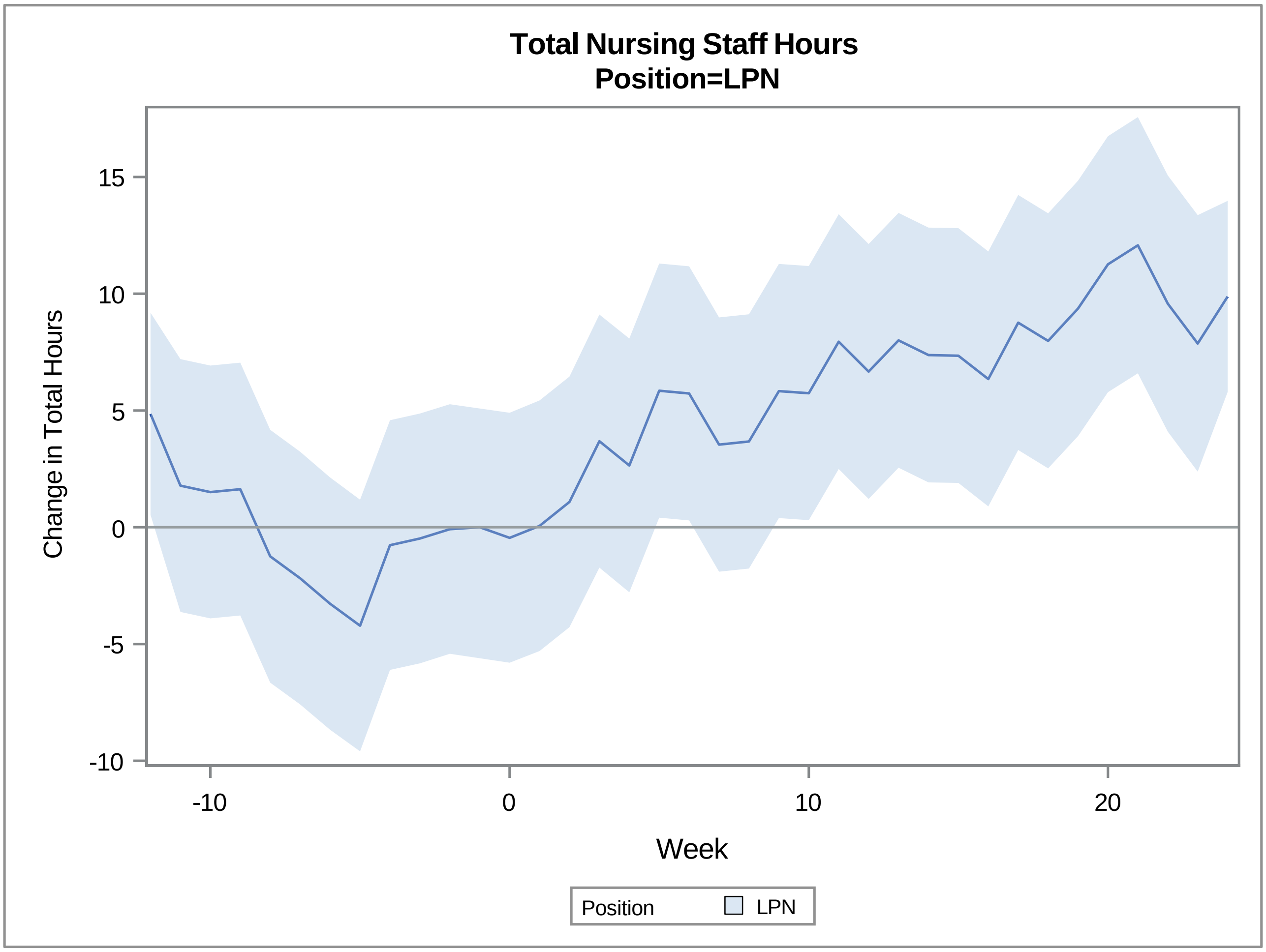

LPN= Licensed Practical Nurse; PPP= Paycheck Protection Program, NH= nursing home

© 2023 Travers JL et al. *JAMA Network Open.*

eFigure5. Change in total hours per week for RNs pre- and post- PPP loan compared to non-PPP NH loan recipients

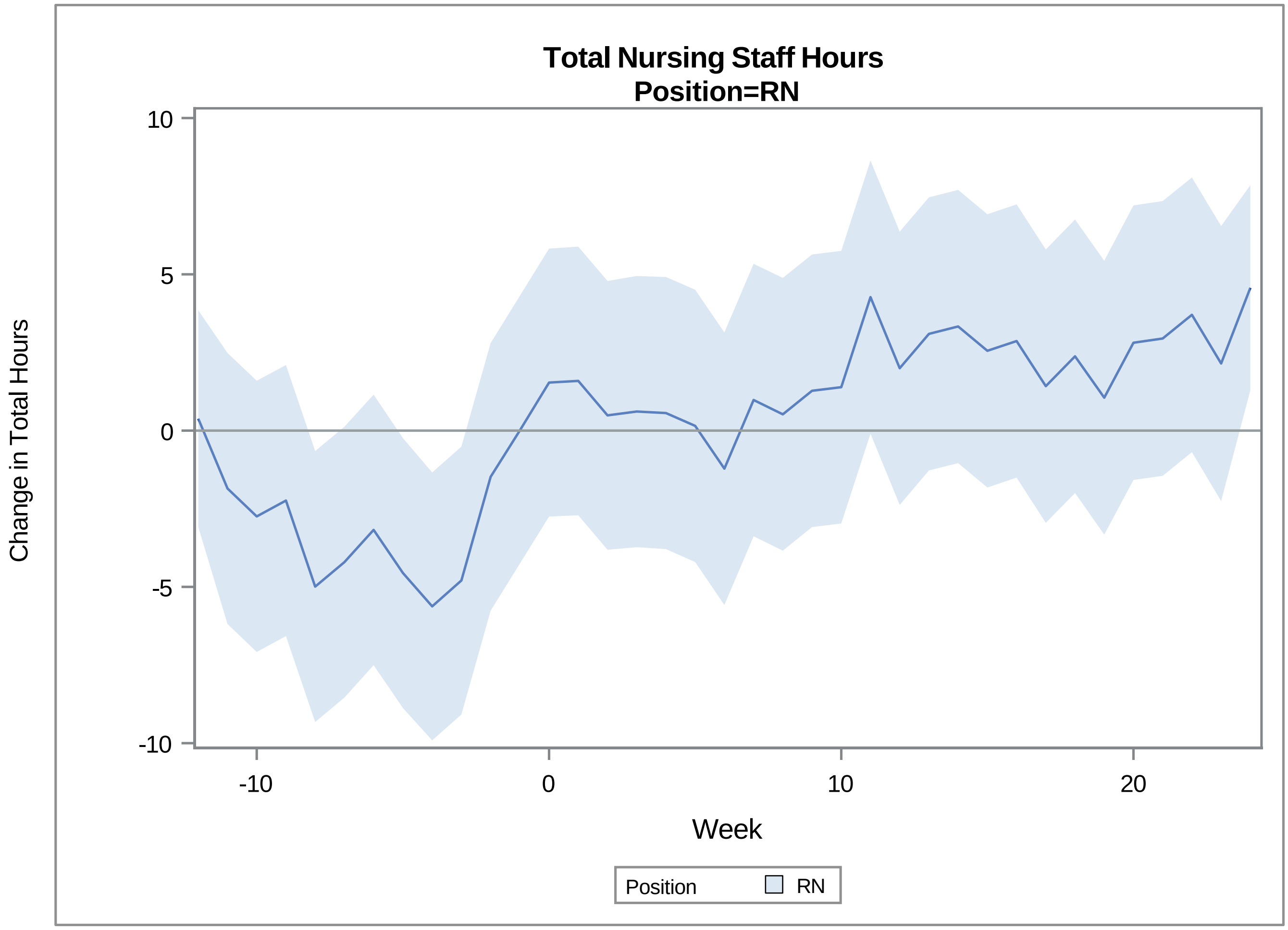

RN= Registered Nurse; PPP= Paycheck Protection Program, NH= nursing home

© 2023 Travers JL et al. *JAMA Network Open.*
