# Supplementary material for: Association of Receipt of Paycheck Protection Program Loans With Staffing Patterns Among US Nursing Homes
Source: JAMA Netw Open. 2023 Jul 27;6(7):e2326122. doi: 10.1001/jamanetworkopen.2023.26122 (PMC10375300; doi:10.1001/jamanetworkopen.2023.26122)
Supplement: Supplement 2. — Data Sharing Statement [file jamanetwopen-e2326122-s002.pdf]

Data Sharing Statement

Travers. Association of Receipt of Paycheck Protection Program Loans With Staffing Patterns Among US Nursing Homes. *JAMA Netw Open*. Published July 27, 2023. doi:10.1001/jamanetworkopen.2023.26122

Data
Data available: Yes
Data types: Deidentified participant data
How to access data: Request to authors
When available: With publication

Supporting Documents
Document types: None

Additional Information
Who can access the data: anyone requesting the data
Types of analyses: for any purpose
Mechanisms of data availability: without investigator support
